# Arduino-Based Myoelectric Control: Towards Longitudinal Study of Prosthesis Use

**DOI:** 10.3390/s21030763

**Published:** 2021-01-24

**Authors:** Hancong Wu, Matthew Dyson, Kianoush Nazarpour

**Affiliations:** 1 School of Informatics, The University of Edinburgh, Edinburgh EH8 9YL, UK; 2 School of Engineering, Newcastle University, Newcastle upon Tyne NE1 7RU, UK; matthew.dyson@ncl.ac.uk

**Keywords:** surface electromyogram, prosthesis control, wearable, low-cost

## Abstract

Understanding how upper-limb prostheses are used in daily life helps to improve the design and robustness of prosthesis control algorithms and prosthetic components. However, only a very small fraction of published research includes prosthesis use in community settings. The cost, limited battery life, and poor generalisation may be the main reasons limiting the implementation of home-based applications. In this work, we introduce the design of a cost-effective Arduino-based myoelectric control system with wearable electromyogram (EMG) sensors. The design considerations focused on home studies, so the robustness, user-friendly control adjustments, and user supports were the main concerns. Three control algorithms, namely, direct control, abstract control, and linear discriminant analysis (LDA) classification, were implemented in the system. In this paper, we will share our design principles and report the robustness of the system in continuous operation in the laboratory. In addition, we will show a first real-time implementation of the abstract decoder for prosthesis control with an able-bodied participant.

## 1. Introduction

Myoelectric prostheses have the potential to restore the functionality of missing limbs. First developed in 1948 [1], myoelectric prostheses are now widely used by amputees in their daily lives. Although research and development have led to significant technological advances in the laboratory, conventional control algorithms are still the mainstream in clinical trials and commercialised applications. A literature search shows that among the 1716 articles that mention daily use of prostheses since 1990, only 69 include the original experiments outside the laboratory [2]. Limited beyond-the-laboratory testing might explain why the prosthesis rejection rate has remained as high as 50% [3,4].

One main reason for the status quo is that, in community settings, advanced prosthetic control algorithms do not perform as well as they do in the laboratory [5]. Factors such as electrode displacement [6,7], changes in limb positions [8,9], extra load on the limb [10], time between adaptation and application [11], and user learning [12,13] can degrade the performance of the prosthesis. Expansion of the training dataset helps to increase the robustness of the control, but training data in community settings are limited, and the data collected in laboratory settings can be different from user behaviour in daily life. In addition, the recalibration procedures for advanced algorithms are more complicated than the conventional control methods, and therefore, it will be challenging, if not impossible, to retune these algorithms in home trials without the support of specialists [14,15].

The limitations of portable devices are another barrier to the translational studies of advanced prosthetic technology. Currently, most of the home trials for prosthesis studies were based on commercialised prosthetic devices [16,17,18]. These products included self-contained control systems in their embedded microcontrollers so that they could plug and play directly in the experiments. Having the merits of convenience and robustness, these systems have limited functions and flexibility. For instance, they relied on basic prosthesis control methods, such as on–off or direct control [19], and the users could not change settings during use [20,21]. Add-on controller kits [22,23] provide platforms for the implementation of advanced control algorithms. Due to the high costs involved and the constraints on adaptable prostheses, users in developing countries have limited access to these devices.

Arduino is an open-source computing platform that can integrate data collection, signal analysis, and control on a single microcontroller. It has different series to match various peripheral devices, and the users can develop systems on built-in libraries. Preliminary studies have reported development of prosthetic control systems on Arduino boards [22,24]. With an open-source integrated development environment (IDE), developers can build the systems easily on the basis of previous designs and utilise modules developed in other projects [25,26,27]. Currently, the Arduino-based prosthesis research still focuses on applications with basic control functions [28,29].

In this paper, we describe the development of a cost-effective myoelectric control system for achieving multiple hand tasks with selectable control algorithms. We demonstrate its functionality and portability and show its feasibility for long-term prosthetic control and fair comparison between control algorithms. The features proposed in this work include the following: (a) a robust system structure that guarantees the accuracy of control signals during long-term operation; (b) the implementation of a clinical controller, human-learning-based controller, and pattern-recognition-based controller on a single system, which allows fair comparisons in prosthetic studies; (c) a friendly user interface that helps users to learn the system control and to adjust the control preferences in take-home studies; and (d) extendable data storage for hand-task recording with time stamps. To guarantee the generalisation, the system was developed on off-the-shelf sensors and development boards (Arduino).

## 2. Methods

### 2.1. System Features

#### 2.1.1. System Overview

**Figure 1:**
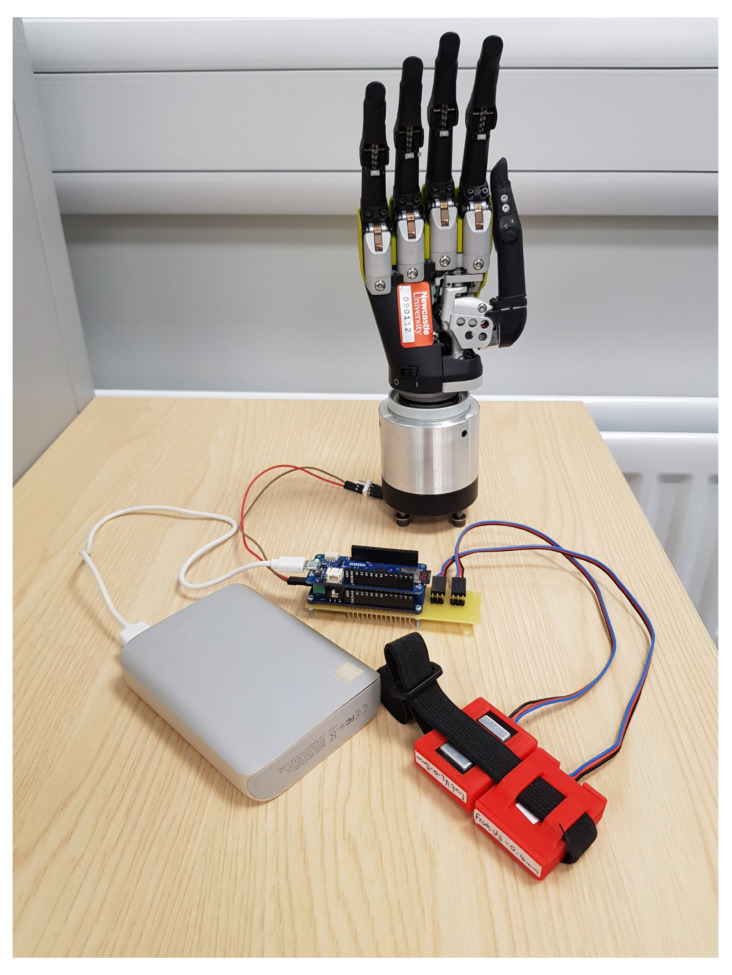
The prototype of the proposed myoelectric control system.

The control system consists of five major components (Figure 1). These are: (1) two sensors for surface electromyogram (EMG) recording (Gravity analog EMG sensors; OYMotion, China); (2) a development board for EMG signal processing, feature extraction, and control command generation (Arduino MKR ZERO; Arduino LLC, USA); (3) a connector module for device communication (Arduino MKR CAN shield; Arduino LLC, USA); (4) a prosthetic hand with six degrees of freedom (DoFs) (Robo-limb hand; Össur, Iceland); and (5) a data-recording unit (32-GB Kingston micro-SD card; Kingston Technology, USA). The Gravity analog EMG sensors amplify the surface EMG signals 1000 times and depress noises through differential input and an analog filter circuit. The amplified EMG signal is sampled by a 10-bit analog-to-digital converter (ADC) through the Arduino analog inputs at a 500 Hz sampling frequency. After processing the EMG signals, the corresponding motor commands are transmitted to the prosthetic hand to drive the motors through the Controller Area Network (CAN) communication.

One reason to develop the system on Arduino is because of its compatibility with a variety of analog front ends and radio frequency (RF) front ends, which is very useful in bridging the EMG sensors with prosthetic devices. It can independently power and interface with varies types of EMG sensors, including MyoWare muscle sensors (Sparkfun, USA), the Grove EMG detector (Seeed Technology Inc., China), and Gravity analog EMG sensors (OYMotion, China). Control signals can be sent to different prosthetic devices, such as the Robo-limb prosthetic hand (Össur, Iceland) and the COVVI hand (COVVI Ltd, UK) through a CAN bus or Bluetooth communication. We selected the Gravity analog EMG sensors in this study because their dry electrode design was simple to use and was more robust in long-term studies and real-world environments [30,31].

#### 2.1.2. Signal Recording and Pre-Processing

To accurately control the prosthesis, the system should record and process the EMG signals quickly so that the delay between muscle contraction and hand movement can be minimised. Our system applied a 500 Hz sampling rate for signal recording. The sampled signals were filtered by an EMG filter module developed by OYMotion (OYMotion, Shanghai, China), which was designed to work synergistically with the analog filters on the Gravity analog EMG sensors. It was implemented by cascading an anti-hum notch filter, a second-order Butterworth lowpass filter, and a second-order Butterworth highpass filter to filter out 50 Hz power line noise, noise above 150 Hz, and noise below 20 Hz, respectively. Considering the variation in muscle strength among users, a calibration module was added to normalise the signals based on individual comfortable contraction levels [32].

#### 2.1.3. User-Friendly Control Interface

**Figure 2:**
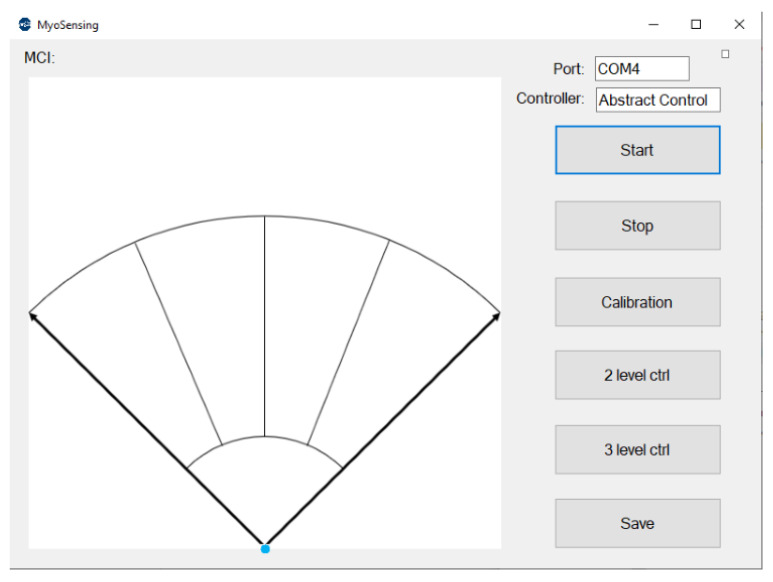
User interface for the proposed system.

A customised control interface was developed on a personal computer (PC) to support users in learning myoelectric control and updating control parameters. As can be seen in Figure 2, the interface is composed of a data visualisation unit and a control panel. The data visualisation unit displays the on-the-fly muscle contraction levels or selected features on the screen. This module helps users develop their control patterns for human-learning-based controllers [33], and it may also support data collection for pattern recognition [34]. The control panel allows users to select the controller based on their unique preferences and to recalibrate the system at home. Recalibration requires the users to rest their muscles and then contract them sequentially at a comfortable contraction level within ten seconds. The EMG signals in the system will be normalised by the calibration data through a normalisation equation [32]. This design aims to simplify the protocols for prosthesis adjustments so that the disruption to the user’s daily life can be minimised.

### 2.2. Controller Modules

Three control algorithms, including the direct control, abstract control, and linear discriminant analysis (LDA) classification, were implemented in the system using two EMG channels. For each channel, the control signal was extracted by the mean absolute value (MAV) of the EMG signals over a 760 ms window with an update rate of 50 Hz. The window size was selected to smooth the control output and maintain an acceptable effector movement [33].

#### 2.2.1. Direct Controller

Direct control is a conventional myoelectric control method that generates a single command (hand open, hand close, or grip switching) at a time [12]. It normally applies threshold-based criteria to determine the activation of control signals [35]. In our two-channel direct controller, the activation of a single control signal directed the prosthetic hand to open and close, respectively. The co-activation of both control signals allowed the user to switch the hand function between power grip, tripod grip, and lateral grip. The hand gesture persisted when neither channel was activated.

#### 2.2.2. LDA Classifier

Classification is the most popular method in myoelectric control. It utilises the training data to develop a classifier that maps the control signals extracted from the input signals onto a discrete output variable that encodes prosthetic activities [36,37]. Because of the robustness, simplicity of implementation, and ease of training, LDA classifiers have frequently been used in previous studies [38].

In this study, the linear discriminant coefficient matrix *W* was trained on a PC to estimate the posterior probability of grips that the user intended to select based on two EMG channels. The trained matrix was uploaded to the system for real-time classification using y=Wx, where *x* is the <2×1> dimensional vector for the MAVs and *y* is the <5×1> dimensional vector encoding the posterior probability. The grip with the highest posterior probability was considered as the output of the classifier.

#### 2.2.3. Abstract Controller

Abstract control is a relatively novel approach for myoelectric control [33]. The mechanism of abstract control is based on the fact that the motor system is able to learn multiple novel muscle synergies to achieve specific motor goals [32,39]. To facilitate the learning, a myoelectric–computer interface (MCI) is used to map the muscle activities to a non-representational multidimensional control space (Figure 2), which provides continuous visual feedback for motor system adaption [40]. The position of the cursor (blue dot in Figure 2) is controlled by the features of the EMG signals, such as the MAV. Users are asked to practise cursor control in a centre-out task to develop abstract muscle synergies [41], and each task will be mapped to a grip or a command of the prosthetic hand. More discussions of how the MCI relates to learning prosthesis control can be found in our previous studies [13,32]. In this research, the cursor position was controlled by the MAVs of two channels, and the MCI was separated into five sections, which were mapped to five different prosthetic activities. When the cursor stayed at the basket (target 0), the prosthetic hand persisted at the previous grip. Moving the cursor from the basket to target 1 to 4 (from left to right) changed the hand to a power grip, tripod grip, lateral grip, or open hand, respectively.

### 2.3. System Evaluation

Ethical approval was granted by the local ethics committee at Newcastle University (Ref: 17-NAZ-056). To show the feasibility of our cost-effective control system, an online analysis of the control signals and a simple pick-and-place experiment were conducted with an able-bodied participant. The participant read an information sheet and gave written consent prior to the experimental sessions. Sensors were connected to the participant’s flexor carpi radialis (FCR) and extensor carpi radialis (ECR) muscles of the forearm to record the EMG activity. To connect the sensor, the skin was cleaned before the experiment. The participant sat on a chair with his elbow near his body and held his forearm at a horizontal position. We found the belly of the ECR and the FCR by asking the participant to repetitively flex and extend the wrist at a comfortable level. After fixing the sensors on the belly with an elastic band, we marked the sensor locations and orientations to ensure that they were at the same position in the multi-day experiment.

For training, the system was connected to the PC user interface shown in Figure 2 through USB, which allowed the participant to control the cursor in the MCI through muscle contraction. The participant first performed a series of contractions to learn the control of the cursor. To be specific, he was required to develop a repeatable muscle strategy that could move the cursor from the basket to the desired targets. This muscle strategy was used for the control of the direct controller and the abstract controller. To train the LDA classifier, the participant was asked to move the cursor to different targets and hold the cursor position within one target for six seconds. The MAVs during the holding period were considered as the patterns for one grip so that the dissimilarity between patterns for different grips could be guaranteed. After training the participant and the classifier, the system was connected to the prosthetic hand, and the participant started to control the hand instead of the cursor.

We first evaluated the controllability and the effector movement by analysing the real-time control signal. The participant tried to make grasps sequentially with all possible grips and to open the hand between two grasps. The MAVs, equivalent cursor positions, controller states, and the selected grips were recorded.

**Figure 3:**
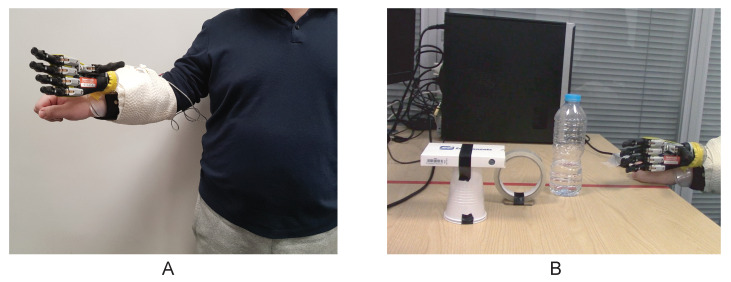
The pick-and-place experiment setup. (**A**) The participant wore the Robo-limb prosthetic hand on his right arm with a customised socket. The prosthetic hand was powered by the battery mounted on the socket and the Arduino system was connected to the computer so that the user could switch the controller during use through the user interface. (**B**) The participant was instructed to grasp and relocate three objects using the prosthetic hand.

To evaluate the feasibility of the system in completing daily activities, the participant fitted the prosthetic hand on his right arm (Figure 3A) to carry out a pick-and-place experiment. Three objects, including a bottle, a roll tape, and a credit card simulator, were placed on a computer desk in front of the participant (Figure 3B). The participant was asked to lift the objects with a power grip, tripod grip, and lateral grip, respectively, and place them on the right-hand side of the red line by opening the hand. Videos of the experiment were recorded and are supplied as supplementary material.

To verify the system performance during a long-term study, we recorded and compared the control signals before and after continuous operation. The myoelectric control system was loaded onto the Arduino board at the beginning of the experiment for a five-day test. On each day, the participant switched on and recalibrated the system based on the calibration protocol. A trial was conducted after the recalibration to ensure that the participant could easily move the cursor from the basket to each target. We kept the system working for eight hours, and the participant was required to repeat the trial at the end of the day. The MAV signals and the cursor traces were recorded and compared.

## 3. Results

### 3.1. Control Signal Analysis

**Figure 4:**
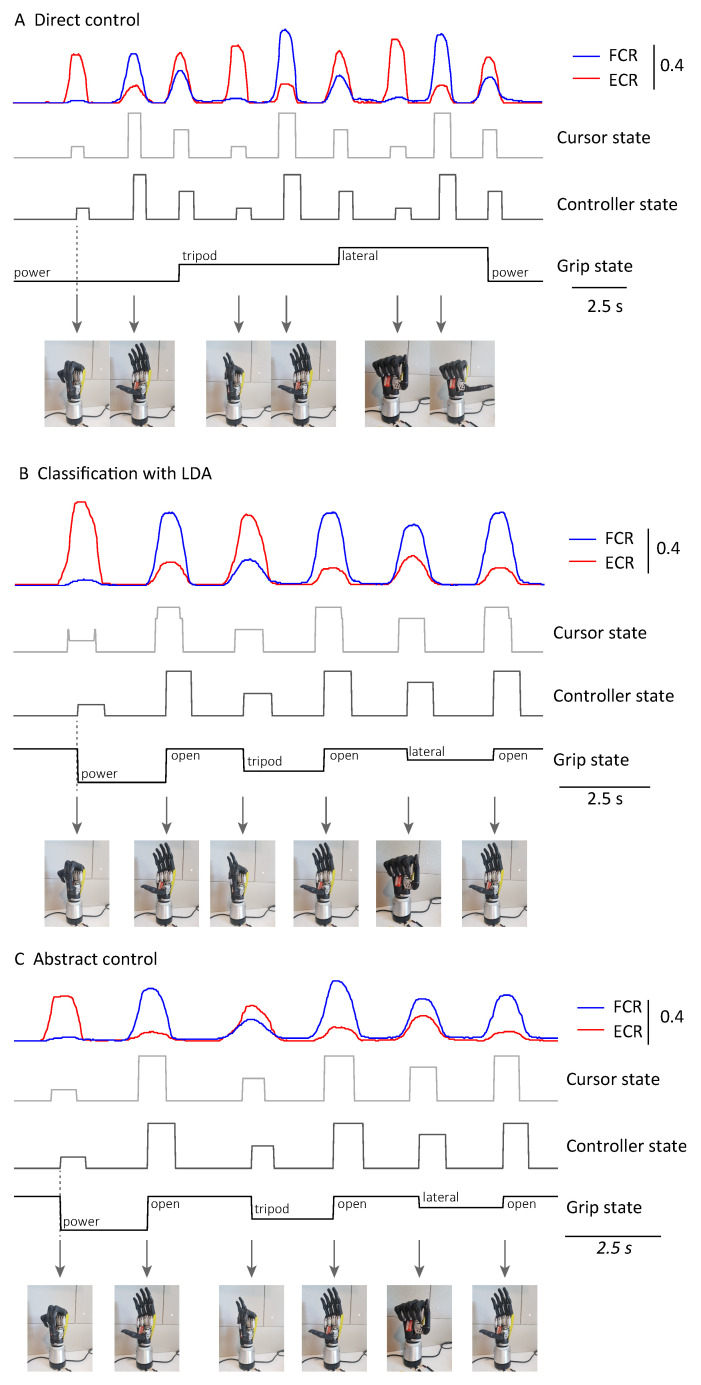
Control signals for the (**A**) direct controller, (**B**) linear discriminant analysis (LDA) classifier, and (**C**) abstract controller.

Samples of the control signals and the corresponding prosthetic hand movements are presented in Figure 4. These demonstrate the relationship between the MAV and state machines within the system.

In the direct control, contracting the FCR and ECR individually moved the cursor from the basket to target 1 and target 4, respectively, which closed and opened the prosthetic hand with the selected grip. Co-contracting the muscles from resting switched the grip selection, and the hand was reset to the pre-set position. In the abstract control and the LDA classification, a grasp was completed by directly selecting the desired grip through muscle contraction/activity. Once a grip was selected, the system reset the hand to the pre-set position and then completed the grasp after a short waiting period.

Because of the continuous updating, the changes in EMG started to take effect on the MAV even though we applied a 760 ms averaging window. The delay was calculated between the time point when the MAV exceeded 5% of the maximum voluntary contraction and the time point when the cursor state changed. Based on this criterion, the average time from the participant’s intention for a grasp until the cursor reached the desired target was 224 ms. The delays for the LDA classification and the abstract control were slightly longer, which were 262 ms and 268 ms, respectively. There was a 240 ms dwell time between the changes in cursor state and the controller state, which required the participant to retain the cursor within the target to make a grasp. The aim of this design was to avoid unintended hand activities due to false triggering or unconscious muscle contraction (Figure 4B).

### 3.2. Pick-and-Place Experiment

**Figure 5:**
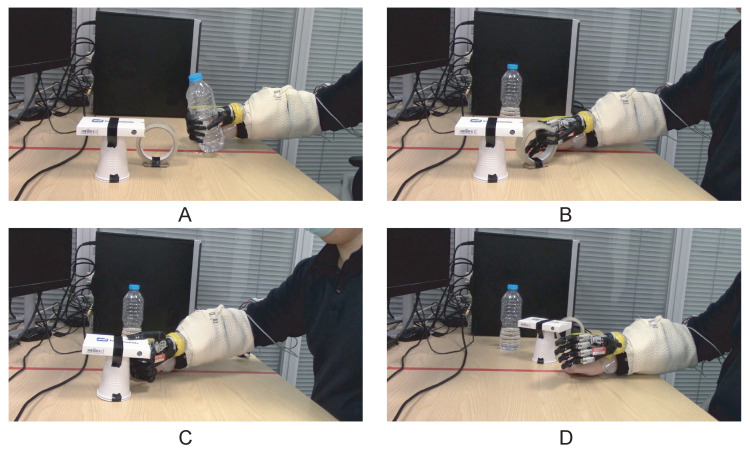
Real-time pick-and-place experiment with three different control algorithms. The participant (**A**) lifted the bottle with a power grip, (**B**) lifted the roll of tape with a tripod grip, (**C**) lifted the credit card simulator with a lateral grip, and (**D**) opened the prosthetic hand to relocate the objects.

The time series of the pick-and-place experiment are illustrated in Figure 5. The prosthetic hand was preset to the open-hand position at the beginning of the test. The participant sequentially moved the bottle, the roll of tape, and the credit card simulator with the corresponding grips, and opened the hand again to finish the test. The real-time performance results for the direct controller, the abstract controller, and the LDA classifier are shown in supplementary material. It took the participant 23 s to complete the test with the abstract controller and the LDA classifier, and it required 28 s with the direct controller.

### 3.3. System Performance in a Day-Long Study

**Figure 6:**
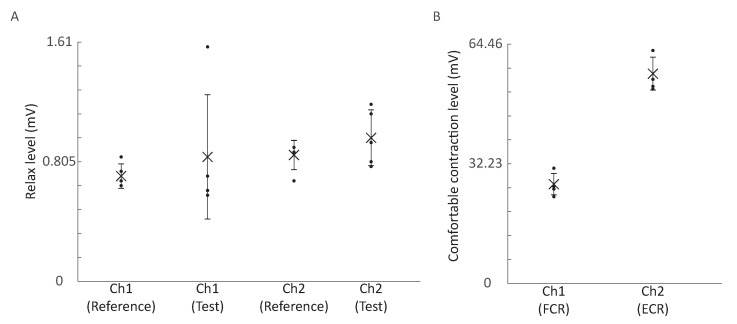
Mean absolute value (MAV) comparisons. (**A**) Comparison of the relaxed levels in the calibration (reference) and at the end of the day (test). (**B**) The comfortable contraction levels for two channels. Cross: mean; error bar: standard deviation; dot: individual data points (n = 5).

The baselines of the control signals are presented in Figure 6A. Continuous system operation did not introduce obvious zero drift to the MAV for the filtered EMG signals. The average MAVs of the two channels at the relaxed level were increased by 0.13 and 0.12 mV, respectively, which was much smaller than the muscle contraction levels in Figure 6B. Possible reasons for the outlier in Ch1 (Test) include the sensor position and the contact between the skin and electrode.

**Figure 7:**
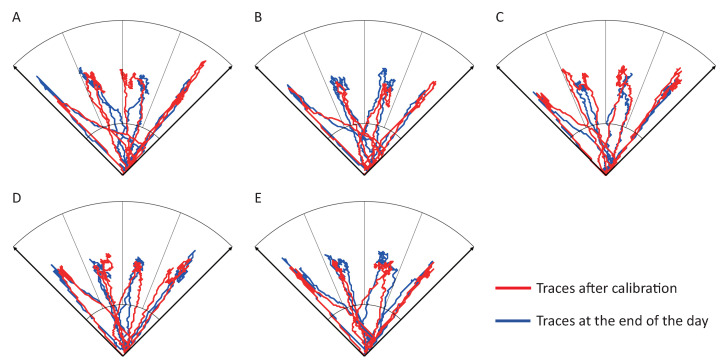
Representative cursor traces on (**A**) Day 1, (**B**) Day 2, (**C**) Day3, (**D**) Day 4, and (**E**) Day 5.

Figure 7 compares the cursor movement between the trial after calibration and the trial at the end of the day. No significant differences can be observed at the baseline of the cursor. The sensitivity of both sensors remained unchanged so that the participant could repeat the cursor trace without recalibration. Some user habits, such as contacting the FCR while the participant was trying to relax the ECR, could be observed in both trials.

## 4. Discussion

We have shown the development of an Arduino-based myoelectric control system with three independent controllers for achieving multiple hand tasks. The system can be realised with a total cost of GBP 120, and the sensors can be replaced with any of their equivalents. The muscle contraction signals were collected by wearable EMG sensors with a 500 Hz sampling rate and could be processed within 20 ms, which allowed the system to provide movement updates in real time.

Considering the portability, we soldered the Arduino boards and connectors to a single printed circuit board (PCB) and removed unnecessary cables in the Gravity analog EMG sensors when designing the prototype (Figure 1). The total weight of the modified system was 67.8 g, which would not add significant load to an arm compared to the weight of a prosthetic limb. The sizes of the PCB and the EMG sensor are 96 × 29 × 29 mm (length × width × height) and 38 × 32 × 11 mm, respectively. We 3D-printed wearable shells for the PCB and the sensors (the red casing in Figure 1) so they could be worn on the arm with straps. When both sensors are actively measuring, the system consumes 16 mA at 3.7 V. Therefore, a rechargeable LiPo battery with 1000 mAh capacity will allow the system to work continuously for more than 48 h, in theory.

Regarding the effector movement, three controllers had similar delays. This meant that the latency was mainly introduced by filtering and the analysis window [42]. With a 50 Hz update rate, the additional latency introduced by the analysis window was below 300 ms and could not be perceivable by the user [43]. We introduced the dwell time to avoid false triggering, but the period was adjustable. It may be reduced for an experienced prosthesis user who is less likely to generate unintended commands. The controller delay of the direct controller was slightly shorter than that of the abstract controller and LDA classifier. One possible reason is that the difficulty of retaining the cursor on the MCI target is higher than for the direct control. This latency may be reduced by systematic training [13]. Meanwhile, it was noticed that the time to achieve three grips with the direct control was longer than those with the LDA classification and the abstract control in Figure 4. Extra pre-setting time for grip switching makes the direct controller effective only for tasks with a small number of grips. Otherwise, the grip switching would be time-consuming [44], which was identified as inadequate for task completion in previous surveys [45].

In the proposed system, the three controllers shared the same EMG sensors, signal processing and feature extraction modules, prosthetic hand drivers, and MCI. This approach allows a fair comparison between the basic performances of the controllers in achieving physical tasks. It also provides a platform to study the differences and similarities between the control of the cursor and the control of the prosthetic hand. For example, it can be observed from the supplementary videos that the extra time of completion with the direct controller is mainly because of the pre-setting time, which takes about one and a half seconds to switch the hand from one grip to the pre-set position of the next grip. The result matches the control signals in Figure 4. It shows the advantages of the abstract control and the LDA classification, and it may also verify that the scores of a controller in cursor control studies [46] can reflect its performances in physical task experiments.

A user interface was developed to visualise the control signals and send commands to the system through a desktop. It allows users to set up the system properly with visual feedback and to conduct recalibration in take-home studies. As learning the control on the MCI increases the performance [13,47], the user interface provides a platform for users to train themselves during long-term home studies. In the current version, the training of the LDA classifier requires the support of MATLAB. Planned future works include embedding the training algorithms into the interface.

To study long-term prosthesis use, system stability is an important aspect. We have shown that the system could provide stable control signals in a five-day test. Daily recalibration of the system only required the participant to provide his relaxed levels and comfortable contraction levels for each muscle for 10 s. Retraining of the LDA classifier was not necessary, as we introduced human learning [48] in the design of the patterns. Instead of retraining the system with new data, which might require several hours with the support of experienced professionals [49,50], the participant could actively map his muscle activities to the patterns.

In addition to the amount of time for which the device is turned on, the system allows the storage of EMG features and control signals, as well as the use of grips to describe prosthetic use. The data can be saved to a PC through the user interface and the on-board micro-SD card. Considering the limitation of storage space, the micro-SD only saves the use of grips with time stamps when new commands have been sent to the prosthetic hand. One possible way to overcome this barrier is the internet of things (IoT) [51,52], which allows the end devices (the proposed system) to upload data to a cloud server, such as the Arduino IoT Cloud. This not only reduces the risks that come with having a large amount of data stored in a single database, but also enables the comprehensive recording of prosthesis use in take-home studies [53], which will be useful in the analysis of prosthesis abandonment.

In this study, the system was only tested on a single able-bodied participant. However, since the EMG sensors are wearable and both the system and the prosthetic hand can be powered by batteries, it can be easily expanded into a wearable device for different-limbed users by mounting the components on the socket. Moreover, the update rate of the system is adjustable, so it is possible to increase the number of EMG channels by allowing a longer time for signal processing and command updates.

## 5. Conclusions

We introduce a system that offers the possibility of longitudinal experiments with advanced prostheses. We implemented it on a cost-effective Arduino-system platform and coupled it with wearable EMG sensors. The system allows users to adjust system settings, learn the control strategy, and record the prosthetic use outside the laboratory. This system enabled fair and balanced comparisons to be conducted between three different control algorithms. Home trials with people with limb differences will be carried out in planned future works.

## Data Availability

The data that support the findings of this study are openly available in Edinburgh DataShare at https://doi.org/10.7488/ds/2977.

## Supplementary Materials

The following are available at https://www.mdpi.com/1424-8220/21/3/763/s1.
